# Redefining Risk Stratification and Endpoints for Clinical Trials in Kidney Transplantation: Rationale and Methodology of Proposals Submitted to the European Medicines Agency by the European Society for Organ Transplantation

**DOI:** 10.3389/ti.2021.10142

**Published:** 2022-05-20

**Authors:** Maarten Naesens, Stefan Schneeberger, the ESOT Working Group, Maarten Naesens

**Affiliations:** ^1^ Department of Microbiology, Immunology and Transplantation, KU Leuven, Leuven, Belgium; ^2^ Department of General, Transplant and Thoracic Surgery, Medical University of Innsbruck, Innsbruck, Austria

**Keywords:** kidney transplantation outcome, EMA guideline, efficacy endpoint, long-term outcome, improvement, European Society for Organ Transplantation, risk stratification

## Abstract

The European Society for Organ Transplantation (ESOT) submitted a Broad Scientific Advice request to the European Medicines Agency (EMA) in 2018, to explore whether updating guidelines on clinical trial endpoints would encourage innovations in kidney transplantation research, thereby improving long-term outcomes for allograft recipients. The request was refined collaboratively by the EMA and ESOT, with the EMA issuing a final response in December 2020. This *Transplant International* special issue explores the topics that were the focus of these interactions between the EMA and ESOT. Articles explore the current issues and dilemmas in kidney transplantation, primarily relating to unclear or outdated risk stratification and markers of transplantation success, although several potential improvements for outcomes assessment are also suggested. Discussions between the EMA and ESOT and recommendations are summarized, in the hope that this project will generate further discussion eventually generating a consensus on clinical trial endpoints and risk stratification, increase the quality of research in transplantation medicine, and improve long-term outcomes for kidney transplant recipients.

## Introduction

Over many decades, progress in the treatment of acute rejection markedly improved the short-term success of kidney allografts, such that graft survival in the first year after transplantation now exceeds 90% [1]. However, improvement rates have decelerated. Data from 135 kidney transplant centers in 21 European countries (187,787 individual transplantations) indicate that the improvement of graft survival has slowed significantly since 2000, even when considering the increased age of donors and recipients [1]. Initiatives to further improve graft survival rates are therefore needed, but the nature of such initiatives is an important point for discussion.

Certainly, several pharmaceutical regimens have been developed, such as efficacious and relatively well-tolerated immunosuppressants, which have enabled very good short-term outcomes to be achieved in patients (and with an acceptable risk of graft rejection). However, the ensuing misconception is that all major hurdles in transplantation have been overcome [2, 3]. Good short-term outcomes that are observed in transplantation recipients do not always translate into satisfactory long-term graft functioning or patient-survival rates [4, 5]. Lack of long-term success creates difficulty in defining suitable surrogate endpoints for clinical trials, which is problematic for ongoing research and could discourage academic/commercial investment in kidney transplantation [3].

Consequently, while clinical progress in kidney transplantation is slowing down, rates of allograft loss continue to be unacceptably high. The extensive negative impact that this has on patient health and well-being—as well as the high long-term health-associated cost burden [6]—clearly indicates a need for novel, effective management strategies, tested according to endpoints that suit current practice and regulations. In addition, there are no approved surrogate markers for long-term graft failure in kidney transplantation, necessitating long-term interventional studies as an urgent priority.

Innovations in kidney transplantation often focus on the prevention and treatment of acute allograft rejection [7]. While current immunosuppressive regimens have reduced the incidence of rejection in low-risk organ recipients [8], many patients have a high immunological risk and could benefit from better preventive and therapeutic options than those available [9]. Stratification of patients and allografts according to immunological risk, however, is not standardized.

## Current EMA Guidance on Clinical Studies for Kidney Transplantation

Released in 2008, the European Medicines Agency (EMA) guideline (CHMP/EWP/263148/06) [10] provides guidance on the conduct of clinical studies for solid organ transplantation (not specific for kidney transplantation) by defining treatment goals, study designs, outcome measures, and data analyses for new immunosuppressive therapies developed to prevent and treat allograft rejection. The guideline [10] defines the primary efficacy endpoint for novel immunosuppressants in solid organ transplantation (not specific for kidney transplantation) as a composite of four outcomes:

• Patient death
• Graft failure—defined by discrete criteria (e.g., permanent return to pre-transplantation treatment modality for a specific period)
• Biopsy-proven acute rejection—including pathological grading for the transplant, outcome, treatment, and response
• Graft (dys)function—defined by best available clear-cut and discrete criteria for kidney, lung, and heart transplantations (e.g., measurement of creatinine/inulin clearance for kidney dysfunction).

Components of the composite endpoint could be omitted for several reasons, such as if there is limited sensitivity/specificity for available biomarkers, or limited consensus about the importance of individual risk factors or cut-off values. Factors such as previous early graft loss (because of immunological factors), re-transplantation, human leukocyte antigen (HLA) mismatch, and presence of HLA antibodies are often taken into consideration, depending on the type of transplantation. The guideline also notes that best attempts should be undertaken to define the recipient’s immunological risk at baseline, using categories such as “low/medium/high” or “elevated/non-elevated.” Finally, CHMP/EWP/263148/06 notes that transplantation outcome is also influenced by surgery and comorbidity [10]. Therefore, reasonably validated scales for assessment of global transplantation risk are important and should be reflected in the target population of clinical studies.

## Rationale for Updating the Guidelines

Not only has clinical organ transplantation changed markedly since the publication of CHMP/EWP/263148/06 [10]; the transplant community now observes signs of substantial decline in the rate of clinical innovation in this field. Consequently, ESOT—the umbrella organization under which all European transplant activities are organized—sought to understand whether updating guidelines on clinical trial endpoints might help to encourage kidney transplantation research, since potentially outdated or unclear definitions of risk groups and markers of success or failure might limit investment or innovation in transplantation medicine.

## Methods

The project that ultimately resulted in the Broad Scientific Advice request and the present special issue began in May 2016, when the European Medicines Agency responded positively to ESOT’s request to begin interactions relating to the overall topic. Within ESOT, the project was coordinated from its inception by Professor Maarten Naesens of KU Leuven, Belgium, with contributions by an international group of experts. This panel of volunteers included nephrologists, surgeons, transplant pathologists, epidemiologists, immunologists, and researchers (Box 1). The key events in the development process were:

• September 26, 2017: First workshop meeting at ESOT Barcelona to outline the project, define the core questions, and appoint working group leaders
• 2017–2018: Briefing package for the EMA was prepared by attendees of the workshop in Barcelona, who collaborated *via* telephone/e-mail and personal interaction
• June 11, 2018: Submission of a briefing package to EMA, outlining the scope and core questions put forward by ESOT
• June 20, 2018: Response from EMA and invitation to submit a formal request for Broad Scientific Advice
• 2018–2019: Establishment of five working groups related to the core questions agreed with EMA (Box 2), on which ESOT sought advice
• 2018–2019: Writing of the first drafts of the answers to the questions and searching consensus within the working groups
○ Each working group approached the Centre for Evidence in Transplantation (CET) with specific data extraction requests
○ Information provided by CET was used by each working group at their own discretion to produce draft documents
○ The documents were drafted by the experts within each working group
• September 17, 2019: Workshop at the ESOT congress in Copenhagen: consensus meeting to discuss the conclusions reached by the working groups
• 2019–2020: Finalization of the consensus document and preparation of the official request for Broad Scientific Advice
• June 5, 2020: Submission of the package to request Broad Scientific Advice from CHMP
• June 6–11, 2020: Start of the Scientific Advice Working Party (SAWP) procedure
• July 6–9, 2020: SAWP discussion meeting, at which a list of issues to be addressed by ESOT was adopted
• September 24, 2020: ESOT submitted additional documentation to the SAWP
• September 30, 2020: Virtual discussion meeting between ESOT and the SAWP, addressing the list of issues
• September 28 to October 1, 2020: SAWP agreed on the advice to be given to ESOT
• December 7–10, 2020: adoption of the advice by CHMP and response received by EMA
• 2020–2021: Reformatting of the package submitted to CHMP to fit publication style (in article form and as a positioning paper), to allow widespread dissemination of ESOT’s answers to the questions and the CHMP advice
• 2021: The ESOT position on each question, including any comments from EMA, is summarized in evidence-based articles within this special issue.

BOX 1 **Individuals involved with the EMA-CHMP request for Broad Scientific Advice project, on behalf of the European Society for Organ Transplantation.**

**COORDINATOR**

Maarten Naesens: Department of Microbiology, Immunology and Transplantation, KU Leuven, Leuven, Belgium

**CONTRIBUTORS**

Jan Ulrich Becker: Institute of Pathology, University Hospital Cologne, Cologne, Germany

Maria Irene Bellini: Department of Surgical Sciences, Sapienza University of Rome, Rome, Italy

Oriol Bestard: Department of Nephrology and Kidney Transplantation, Vall d’Hebrón University Hospital, Barcelona, Spain

Georg A. Böhmig: Division of Nephrology and Dialysis, Department of Internal Medicine, Medical University of Vienna, Vienna, Austria

Klemens Budde: Department of Nephrology and Medical Intensive Care, Charité Universitätsmedizin Berlin, Berlin, Germany

Fergus Caskey: Department of Population Health Sciences, University of Bristol, Bristol, United Kingdom

Frans Claas: Eurotransplant Reference Laboratory, Department of Immunology, Leiden University Medical Center, Leiden, Netherlands

Lionel Couzi: Department of Nephrology, Transplantation and Dialysis, Bordeaux University Hospital, Bordeaux, France

Fritz Diekmann: Department of Nephrology and Kidney Transplantation, Hospital Clinic Barcelona, Barcelona, Spain

Fabienne Dobbels: Academic Center for Nursing and Midwifery, Department of Public Health and Primary Care, KU Leuven, Leuven, Belgium

Lucrezia Furian: Kidney and Pancreas Transplantation Unit, University of Padua, Padua, Italy

Denis Glotz: Paris Translational Research Center for Organ Transplantation, Hôpital Saint Louis, Paris, France

Josep Grinyó: Emeritus Professor of Medicine, University of Barcelona, Barcelona, Spain

Uwe Heemann: Department of Nephrology, Technical University of Munich, Munich, Germany

Dennis Hesselink: Erasmus MC Transplant Institute, University Medical Center Rotterdam, Rotterdam, Netherlands

Luuk Hilbrands: Department of Nephrology, Radboud University Medical Center, Nijmegen, Netherlands

Ina Jochmans: Department of Microbiology, Immunology and Transplantation, KU Leuven, Leuven, Belgium

Alexandre Loupy: Paris Translational Research Center for Organ Transplantation, Hôpital Necker, Paris, France

Nizam Mamode: Department of Transplantation, Guy’s and St Thomas’ NHS Foundation Trust, London, United Kingdom

Rainer Oberbauer: Department of Nephrology and Dialysis, Medical University of Vienna, Vienna, Austria

Liset Pengel: Centre for Evidence in Transplantation, Nuffield Department of Surgical Sciences, University of Oxford, Oxford, United Kingdom

Marion Rabant: Department of Pathology, Hôpital Necker-Enfants Malades, Paris, France

Marlies Reinders: Erasmus MC Transplant Institute, Department of Internal Medicine, University Medical Center Rotterdam, Rotterdam, Netherlands

Lionel Rostaing: Department of Nephrology, Dialysis and Transplantation, Toulouse University Hospital, Toulouse, France

Candice Roufosse: Centre for Inflammatory Disease, Department of Immunology and Inflammation, Imperial College London, London, United Kingdom

Stefan Schneeberger: Department of General, Transplant and Thoracic Surgery, Medical University of Innsbruck, Innsbruck, Austria

Daniel Seron: Department of Nephrology and Kidney Transplantation, Vall d’Hebrón University Hospital, Barcelona, Spain

Olivier Thaunat: Department of Transplantation, Nephrology, and Clinical Immunology, Edouard Herriot Hospital, Hospices Civils de Lyon, Lyon, France

Allison Tong: Sydney School of Public Health, The University of Sydney, Sydney, Australia

BOX 2 **Questions presented to EMA by ESOT in their request for Broad Scientific Advice on clinical trial design and endpoints in kidney transplantation. This special issue presents current knowledge and perspectives from the European Society for Organ Transplantation (ESOT) on these five core questions, based on evidence and clinical and research experience.**

Q1: Does CHMP agree with the updated definitions of rejection and their potential use as primary endpoints in studies of kidney transplantation?

Q2: Does the CHMP agree with the proposed definitions of allograft (dys)function in kidney transplantation, and the recommendations for parameters that could be used as primary endpoints in clinical trial settings?

Q3: Does CHMP agree with the proposed specific risk profiles for kidney transplantation which determine background risk of rejection associated with immunosuppressive therapy?

Q4: Does CHMP agree that long-term outcome after kidney transplantation is an area of unmet medical need, for which conditional marketing authorization procedures should be considered, to facilitate timely access to new therapies? If so, does CHMP agree with the proposed surrogate endpoints for clinical trials for therapies requiring conditional marketing authorization?

Q5: Does CHMP agree with the proposed patient reported outcomes as (primary/secondary) endpoints for use in clinical trials of kidney transplantation interventions?

## Conclusion

In June 2020, ESOT presented an extended form of the articles and supporting materials in this special issue to the EMA as one document, as part of the package to request Broad Scientific Advice from CHMP. Following constructive responses from the EMA, this special issue is published to communicate outcomes and extend discussions among the wider kidney transplant community. Ultimately, it is hoped that endpoints suitable for future clinical trials and better consensus on risk stratification are developed and agreed globally, thereby increasing the quality of future research and evidence, and advancing the practical management of kidney transplantation, particularly for long-term outcomes.

## References

[1] Coemans M Süsal C Döhler B Anglicheau D Giral M Bestard O Analyses of the Short- and Long-Term Graft Survival after Kidney Transplantation in Europe between 1986 and 2015. Kidney Int (2018) 94:964–73. 10.1016/j.kint.2018.05.018 30049474

[2] OʼConnell PJ Kuypers DR Mannon RB Abecassis M Chadban SJ Gill JS Clinical Trials for Immunosuppression in Transplantation: the Case for Reform and Change in Direction. Transplantation (2017) 101:1527–34. 10.1097/TP.0000000000001648 28207630

[3] Stegall MD Morris RE Alloway RR Mannon RB. Developing New Immunosuppression for the Next Generation of Transplant Recipients: the Path Forward. Am J Transplant (2016) 16:1094–101. 10.1111/ajt.13582 26730885

[4] Mayrdorfer M Liefeldt L Wu K Rudolph B Zhang Q Friedersdorff F Exploring the Complexity of Death-Censored Kidney Allograft Failure. J Am Soc Nephrol (2021) 32:1513–26. 10.1681/asn.2020081215 33883251 PMC8259637

[5] Ying T Shi B Kelly PJ Pilmore H Clayton PA Chadban SJ Death after Kidney Transplantation: an Analysis by Era and Time post-transplant. J Am Soc Nephrol (2020) 31:2887–99. 10.1681/asn.2020050566 32908001 PMC7790214

[6] Vanholder R Domínguez-Gil B Busic M Cortez-Pinto H Craig JC Jager KJ Organ Donation and Transplantation: a Multi-Stakeholder Call to Action. Nat Rev Nephrol (2021) 17:554–68. 10.1038/s41581-021-00425-3 33953367 PMC8097678

[7] Abramowicz D Oberbauer R Heemann U Viklicky O Peruzzi L Mariat C Recent Advances in Kidney Transplantation: a Viewpoint from the Descartes Advisory Board*. Nephrol Dial Transplant (2018) 33:1699–707. 10.1093/ndt/gfx365 29342289 PMC6168736

[8] Naesens M Thaunat O. BENEFIT of Belatacept: Kidney Transplantation Moves Forward. Nat Rev Nephrol (2016) 12:261–2. 10.1038/nrneph.2016.34 27026349

[9] Bestard O Couzi L Crespo M Kessaris N Thaunat O. Stratifying the Humoral Risk of Candidates to a Solid Organ Transplantation: a Proposal of the ENGAGE Working Group. Transpl Int (2021) 34:1005–18. 10.1111/tri.13874 33786891

[10] European Medicines Agency. Clinical Investigation of Immunosuppressants for Solid Organ Transplantation. CHMP/EWP/263148/06 (2008). Available at: https://www.ema.europa.eu/en/clinical-investigation-immunosuppressants-solid-organ-transplantation (Accessed October 18, 2021).

